# Modelling celullar communication with scale-free networks

**Published:** 2008-04-15

**Authors:** Dobrescu Radu, Purcărea Victor

**Affiliations:** * University Politehnica of Bucharest, Faculty of Automatic Control and Computers; ** ”Carol Davila” University of Medicine and Pharmacy, Bucharest, Romania

**Keywords:** complexity, biological systems, scale free networks, cellular signaling pathways, intra-cellular and inter-cellular communication model

## Abstract

The paper proposes a model that brings to light the characteristics of several complex systems having similar scale-free network architecture. The properties of this kind of network are compared with those of other methods which are specific for studying complex systems: nonlinear dynamics and statistical methods. We place particular emphasis on scale-free network theory and its importance in enhancing the framework for the quantitative study of complex biological systems. The advantages and limits in understanding the structure of cellular signaling networks of this model are finally discussed.

## 1. Introduction 

The science of complexity studies systems with a large number of elements, building blocks or agents, capable of interacting with each other and with their environment. The interaction between elements may occur only with immediate neighbours or with distant ones; the agents can be all identical or different; they may move in space or occupy fixed positions, and can be in one of two states or of multiple states. Nevertheless, the common characteristic of all complex systems is that they display organization without any external organizing principle being applied. The modelling of complex systems is a priority of the world’s scientific community and approaches several solutions, but in a rough sense. The current toolbox involves three main areas: (i) nonlinear dynamics and chaos, (ii) statistical physics, including discrete models, and (iii) network theory.

The stock market, metropolitan cities, metabolic pathways, ecosystems, the Internet or the human brain, are all complex. In the last years many research studies showed that all these systems share similar network architectures. Network theory has become one of the most visible pieces of the body of knowledge that can be applied to the description, analysis, and understanding of complex systems. 

Living systems are complex assemblies of macromolecules which interact in different ways, in the framework of a complicated network. Each component of such a network can influence the global behaviour and consequently the storage and delivery of the whole set of information can be solved only with high performance computing techniques. One can consider that only parallel processing procedures run on supercomputers will allow studying such huge models. More than that, methods and tools for inference of unobservable network performance characteristics and for predicting the behaviour at different scales are necessary in such a large networking environment. A model where inference based on self similarity can be applied is the scale-free network.

The concept of the scale-free network was introduced in the seminal work of Barabási and Albert [**1**], which found that a number of real-world networks have a scale-free degree distribution with tails that decay as a power law. These networks grow by adding new connections to a node with a probability proportional to the number of the existing connections. 

## Characteristics of the scale-free networks

Actually the free-scale network model is a refinement of the *small-world network*, structures proposed by Watts and Strogatz [**2**]. In such networks, the number of intermediaries between any pair of nodes in the network is quite small (property typical for random graphs). Meanwhile, the local redundancy is large (property typical for ordered lattices). Watts and Strogatz probed the structure of their small-world network model via two quantities: (i) the shortest average distance L between all pairs of nodes in the network, and (ii) the average clustering coefficient C of the nodes in the network. For a d-dimensional lattice one has L ~N^1/d and C = O(1), where N is the number of nodes in the network; for a random graph one has L ~lnN and C ~ 1/N. But this model doesn’t take into consideration the degree distribution, i.e., the distribution of number of connections of the nodes in the network – a problem solved by using the scale-free network model.

2.1. General SFN properties 

Barabasi and Albert suggested that scale-free networks emerge in the context of growing network in which new nodes connect preferentially to the most connected nodes already in the network. Specifically,

**Fig. 1:**
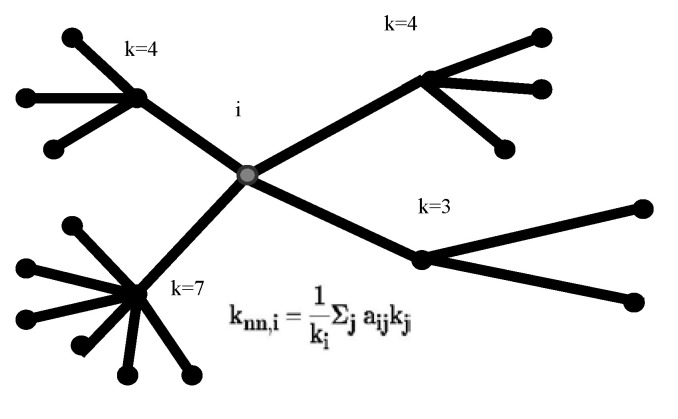
A scale free network graph

where n is the time and number of nodes added to the network, n0 is the number of initial nodes in the network at time zero, ki is the degree of node i and pi(n + 1) is the probability of a new node, added at time n+1 linking to node i.

As illustrated in **Figure 1**, as time passes, the degree distribution of the nodes becomes more and more heterogeneous since the nodes with higher degree are the most likely to be the ones new nodes link to. Significantly, scale-free networks provide extremely efficient communication and navigability as one can easily reach any other node in the network by sending information through the “hubs” - the highly-connected nodes. The efficiency of the scale-free topology and the existence of a simple mechanism leading to the emergence of this topology led many researchers to believe in the *complete* ubiquity of scale-free network. Note that scale-free networks are a subset of all small-world networks because (i) the mean distance between the nodes in the network increases extremely slowly with the size of the network and (ii) the clustering coefficient is larger than for random networks. 

2.2. Diameter of scale-free networks

It was shown that scale-free networks with degree exponent 2<λ<3 possess a diameter D ~ ln ln N, smaller even than that of random and small world networks [**3**]. If the network is fragmented, we will only be interested in the diameter of the largest cluster (assuming there is one). In this study we consider the diameter of a Molloy-Reed scale-free network [**4**] defined as the *average* distance between any two sites on the graph. Actually, it is easier still to focus on the radius of a graph,

L≡〈l〉

as the average distance of all sites from the site of highest degree in the network. The diameter of the graph D is restricted to L≤D ≤2L and thus scales like L.

2.3. Minimal graphs and lower bound

 Cohen, et al., show that the radius of any scale-free graph with λ>2 has a rigorous lower bound that scales as ln ln N [**5**]. The smallest diameter of a graph, of a given degree distribution, is achieved by the following construction: start with the highest degree site, then connect to each successive layer the extant sites of highest degree, until the layer is full. By construction, loops will occur only in the last layer. To bound the radius L of the graph, we will assume that the low degree sites are connected randomly to a giant cluster. On the other hand, if we start uncovering the graph from any site - provided it belongs to the giant component – then within a distance l2 from this site there are at least l2 bonds. Since l= l1+l2, all sites are at a distance of ln ln N from the highest degree site, and L= ln ln N is a rigorous lower bound for the diameter of scale-free networks with λ>2. In a similar way, one can demonstrate that the scaling of D ~ ln ln N is actually realized in the general case of *random* scale-free graphs with 2<λ<3. 

## 3. Recent approaches in biological systems modeling 

Biological systems are complex systems built from a dynamic web of interconnected feedback loops marked by interdependence and redundancy. Complex systems have properties that cannot wholly be understood by understanding the parts of the system. The properties of the system are distinct from the properties of the parts, and they depend on the integrity of the whole. The only real possibility to study such integrated systems is to use mathematical modelling. Mathematical models can serve several distinct purposes. They can be used to analyse experimental results and provide predictions and suggestions for follow-up experiments, or they can attempt to synthesize existing knowledge and provide a theoretical framework for the interpretation of existing paradigms. On the other hand, the more assumptions that have to be put into the model, the harder it is to be confident about the conclusions; but a well designed model can test different assumptions and provide important new insights into questions that cannot be readily answered experimentally.

The evolution in every biodynamic system can be considered as a result of information transfer in a complex cellular/molecular communication system. Although molecular biology is mainly focused on identification of genes and functions of their products, which are components of the system, the major challenge of the research is to understand the biological system within a consistent framework of knowledge built up from the molecular level to the functional system level – not only gene networks, but also protein networks, signalling networks or metabolic networks.

At a very abstract level, a cell can be divided into two general subnetworks, a regulatory network and a metabolic network. The metabolic network is mainly occupied with substance transformation to provide metabolites and cellular structures. The regulatory network’s main task is information processing for the adjustment of enzyme concentrations to the requirements of variable internal and external conditions. This network involves the use of genetic information. **Table 1** presents a short description of the characteristics of such networks.

In our approach the model for intercellular communication is a highly inhomogeneous scale-free network in which a few highly connected cells play a central role in mediating interactions among numerous, less connected cells. To make this model efficient implies hard work on its self-similarity property in order to determine only a few numbers of connections. One possible function of this model is to activate output only if the input signal is persistent and to allow a rapid deactivation when the input goes off. Between each pair of nodes, multiple edges (in both directions) are possible. Also edge weights (at least for the name/type of the connection) are necessary to reflect biological communication in a realistic manner. Note that the number of cell types in the human body is small (approximately 200) and fixed. The number of edges is in contrast of higher magnitude and varies in time. So, one of the challenges for future work will be to deal analytically and explanatory with this kind of complexity. 

**Table 1:** Comparison between cellular networks

Cell Network	Task	Examples
Metabolic pathway	Enzyme reactions on chemical substances	Intermediary / Secondary / Macromolecular Metabolism
Regulatory pathway	Macromolecular interactions. Direct protein-protein interactions and gene expressions	Membrane transport, signal transduction, ligand-receptor interaction, cell cycle, cell death

The complex network of biochemical reaction/transport processes and their biochemical spatial organization make the development of a predictive model of living cell a great challenge. Cell signalling, cell motility, organelle transport, gene transcription and translation, morphogenesis and cellular differentiation cannot easily be accommodated into existing computational frameworks. Conventional approaches using numerical integration of continuous, deterministic rate equations can prove useful when systems are large or when molecular details are of little importance. However when the resolution of experimental techniques increases, conventional models become unwieldy. Difficulties include the importance of spatial location within the cell, the instability associated with reactions between small numbers of molecular species and the combined explosion of large numbers of different species. One of the first methods used to model molecular interactions were the stochastic methods. In the stochastic modelling approach, rate equations are replaced by individual reaction probabilities and the output has a physically realistic stochastic nature. But in the cell, various components interact in diverse ways. All cellular subsystems are highly nonlinear, and subsystem couplings are often nonlinear as well. This nonlinearity indicates that the whole system is not equivalent to the sum of its subsystems. Cell simulators must therefore allow simulation of cell subsystems in both isolated and coupled forms. To simulate coupled subsystems, it is necessary to perform computations on mutually interacting subsystems with different computational properties on a single platform. There is, however, no universal algorithm that can efficiently simulate all subsystems at once, so simulators must allow multiple computation algorithms to coexist in a single model. 

The most realistic approach on the informational flow of signals in biological networks seems to be the separate study of the communication inside the cell (intracellular communication networks) and respectively *intercellular* communication, which links *intracellular* signalling networks of different cells and cell types. 

Signal transduction networks allow cells to perceive changes in the extra cellular environment in order to produce an appropriate response. A cellular process network mediates the transmission of extra cellular signals to their intracellular targets. In general, the external signals are transmitted to the interior of the cells through receptors activating diverse signalling pathways. They can follow a single way and generate an answer or a specific cellular final process, or branch out and give rise to others. These pathways considered as a whole, form an interconnected network, because pathways corresponding to different stimuli cross and generate alternative trajectories. This intracellular signalling implies several molecular processes. The signals can be as simple as the direct introduction of the signal to the nucleus and the activation of the transcription of proteins involved in the specific cellular function, which is expected. On the other hand, they can be very complicated and include multiple stages. For example, the receptor activates effector proteins like second messengers, kinases or phosphatases. They, in turn, activate transcription factor proteins, which determine the transcription of gene coding for proteins involved in the specified cellular function.

Computational models in signal transduction pathways have been constructed using different points of view. Each research group chose the approach which seemed best for them and applied the most adequate computational tool for their purpose. This perspective involves a range of factors, from the types of information processes present at cellular level, such as sequential, parallel, distributed, concurrent and emergent; to the cognitive capabilities exhibited by certain signal transduction pathway components, such as memory, learning, pattern recognition and handling fuzzy data. In this sense, several computational approaches have been proposed to model the cellular signalling pathways, such as artificial neural networks [**6**], Boolean networks [**7**], Petri nets [**8**], rule-based systems [**9**], cellular automata [**10**], and multi-agent systems [**11**]

**Table 2** summarizes the main characteristics of these computational approaches, taking into account the idea behind the approach, the cognitive capabilities that can be modelled, types of present information processing, and the aspect of cellular signalling to be modelled. 

**Table 2:** Characteristics of computational methods

Comp. approach	Characteristics
Boolean networks	The cell can be modeled as a network of two state components interacting between them. The state of each component depends of a particular boolean function.
Expert systems	The interactions (activation, phosphorylation, etc.) between signaling network components are modeled using production rules
Differential-algebraic equations	An ODE equation is built or each molecule x describing its relationship with all relevant molecules y
Cellular automata	The interaction between cells or molecules is modeled as a matrix, where the state of an element of the matrix depends on the states of the neighbouring elements.
Petri nets	The cell is seen as a connected graph with two types of nodes. One type represents elements, such as signaling molecules, the other type represents transitions.
Artificial neural networks	The proteins in signaling networks are seen as artificial neurons in ANN. Like an artificial neuron, a protein receives weighted inputs, produces an output, and has an activation value.
Distributed systems (agents)	The cell is seen as a collection of agents working in parallel. The agents communicate between them through messages.

One can observe that some models are most adequate for intracellular communication and others for intercellular communication. Nevertheless, the models based on scale-free networks have the possibility to integrate different approaches - starting from a set of autonomous agents communicating between them through a shared data structure, where each agent is implemented as a neural network, a Boolean network or a molecular automaton, depending on the complexity of the task carried out by the agent and the knowledge degree or cognitive capabilities required by it.

## 4. Using scale-free networks in cellular communication modeling 

In our approach to model intercellular communication the basic network model consists of cell types as nodes and of intercellular signalling species (first messengers) connecting the nodes. Because communication between cell types occurs in an explicit direction and various kinds of communication might exist, the resulting graph is directed. Between each pair of nodes multiple edges (in both directions) are possible. Also edge weights (at least for the name/type of the connection) are necessary to reflect biological communication in a realistic manner. Thus, we do not model each individual cell, but the principal connections between cell types. In contrast to most other network models investigated recently this *intercellular* network possesses connectivity complexity rather than node complexity. Consequently we are interested in constructing simple connected scale-free graphs having n nodes and a particular graph degree D. It is possible to construct such a graph via a two phase deterministic algorithm that builds the network incrementally using the ordered list of elements in D. In the first phase, one builds a single connected component containing cycles, and in the second phase, any remaining nodes are used to build trees that attach to the most connected component, according to the power-law of connectivity distribution probability.

In order to obtain significant results, we start by considering a set of standard metrics of the model graph (the size and the number of interactions of the network), together with the size of the largest component. An important feature of the graph is the average degree, where the degree of a given node is defined as the number of its connections. The average degree is obtained by <k> = 2l/n with l being the total number of links in the graph and n the number of nodes (the factor 2 takes into account that each link contributes to the degree of two nodes). Another characteristic of the graph’s local cohesiveness is provided by the clustering coefficient, defined for any vertex (node corresponding to the protein i) as the fraction of connected neighbors of i: Ci = 2li / ki (ki-1) where li is the number of links connecting neighbors of i and ki(ki-1)/2 the number of possible connections among neighbors. A more global method for characterizing the graph is the mean clustering coefficient

$$\langle C\rangle =(1/n)\sum_{1}^{n}C_{i}$$

where the average is over all the n proteins in the network. 

The problem to solve is that limited information-processing capabilities have a significant and quantifiable effect on the large-scale structure of growing networks. To test such a network model and to get better insight into intercellular communication we have constructed the model of cell to cell (P2P – peer to peer) communication in a similar way as the tissue-to-tissue communication networks from the CSNDB (Cellular Signaling Network Database [**12**]), one of the few databases providing usable data directly for intercellular communication networks. The information from CSNDB regarding intercellular networks is a list of ligand-receptor interactions and the tissue location of both the ligand and the receptor molecule. From this information one can conclude that tissues sharing a ligand-receptor interaction are connected and establish a communication relation. 

Another important problem is that of the network structure type. There are two classes of P2P networks: unstructured networks with better resilience and structured networks (like SFN) with better performance, especially with fast response time. **Table 3** presents a comparison between these two main classes of networks.

**Table 3:** Comparison between cellular networks

Unstructured Networks	Structured Networks
No specific topology	Predetermined topology
Random connections	Predetermined connections
Offer better resilience to network dynamics (nodes joining and leaving, node failure and network attacks)	Degraded performance during node removals (needs much maintenance), node failures and network attacks
Bad performance, node reachability, response time and no diameter guarantee	Better performance, faster response time and low diameter
Lack of scalability, network partitioning	More scalable, but problem in generic keyword searches
Resilient in attacks	Vulnerable in attacks

The advantages of the model based on a structured scale-free network are as follows:

• In power-law networks many nodes have low degree and few nodes have a very high degree; these high connected nodes act as hubs for the rest nodes

• In our model nodes with degree higher than the average emerge as preferred nodes

• Because every new node that joins the network wants to connect to a preferred node for better visibility, the model guarantees power-law for degree distribution

• Power-law networks have a low diameter and they can grow while maintaining a low diameter (scale-free)

## 5. Conclusions

It is imperative to utilise scale free network as a method for facilitating biological network analysis, at a high abstract level. Such approaches can reveal remarkable characteristics of biological phenomenon undistinguished by non-network based methods.

The need for enhanced computational ability is most evident when one attempts to couple large numbers of individual units into highly interactive and largely parallel networks. The proliferation of information transferred in such networks introduces the need for these systems that provide a framework for classifying information, spatial statistics for analyzing patterns, and dynamic simulation models that allow the integration of information across multiple spatial, temporal, and organizational scales. It is impossible to ignore the apparent universality of pair interactions among the various elements of a complex system. Instead of chance and randomness, one must consider that the system organization is governed by a high degree of internal order. Each node selected in order to be discussed as an element of a network of interacting constituents, implies to locate and quantify the interplay between behaviour, structure and function. It can be approached from the bottom up, moving from cells to modules, or from the top to the bottom, starting from the network’s scale-free and hierarchical nature and moving to specific modules. In either case, it must be acknowledged that structure, topology, network usage, robustness and function are deeply interlinked. 

The edge complexity could be reduced in different respects. For instance the clustering of a network derived from the connectivity and distribution of the nodes might reveal sub-networks of intense communication or the impact of distinct nodes for the whole system. Also the validation of the biological or physical plausibility of scale free networks reconstructed from databases is of major importance. The paper has confirmed that some emergent features are plausibly consistent with the main objective – to offer a model for intercellular communication. Network modelling, quantitative analysis and laboratory experiments have to be combined in various ways to gain new insights.
